# Venezuelan Equine Encephalitis Virus: Structural Biology, Vaccines, and Advances in Functional Antibodies

**DOI:** 10.3390/vaccines14010023

**Published:** 2025-12-24

**Authors:** Rui Tang, Daojing Wang, Guojiang Chen, Chenghua Liu, Liang Zhang, Fenghao Peng, Jijun Yu, Xinying Li, Heng Luo, Yan Wen, Chunxia Qiao

**Affiliations:** 1 College of Biotechnology, Campus of Jiangsu University of Science and Technology, Zhenjiang 212100, China; 241111802124@stu.just.edu.cn (R.T.); luoheng@just.edu.cn (H.L.); 2 State Key Laboratory of National Security Specially Needed Medicines, Beijing 100850, China; 18511167097@163.com (G.C.);

**Keywords:** venezuelan equine encephalitis virus, structure, vaccines, therapeutic antibodies

## Abstract

Background: Venezuelan equine encephalitis virus (VEEV) poses a significant public health and biodefense threat due to periodic epidemics of severe neurological disease in the Americas, yet no licensed human vaccines or specific antiviral therapies exist. Methods: We comprehensively reviewed the current literature across three core domains: structural biology, vaccine development, and therapeutic antibodies. Results: First, we detail the complex viral structural proteome (including E1/E2 glycoproteins and Capsid), focusing on the LDLRAD3 entry receptor interaction. Second, we overview vaccine strategies, covering live-attenuated, VLP, and nucleic acid platforms designed to induce robust neutralizing antibodies. Finally, we examine the evolution of therapeutic antibodies, highlighting that optimal protection often relies on both neutralization and Fc effector functions, particularly for antibodies targeting the fusion loop or receptor-binding sites. Conclusions: Integrating structural insights with advanced antibody and vaccine engineering is essential for establishing effective clinical interventions capable of preventing future outbreaks and treating infected individuals.

## 1. Introduction

Classified within the genus *Alphavirus* of the family *Togaviridae*, Venezuelan equine encephalitis virus (VEEV) represents a complex of small, phylogenetically related viruses possessing positive-sense RNA genomes [1]. As an important re-emerging arbovirus, VEEV poses a continuous threat to public health and the veterinary health of equids [2]. The virus was first successfully isolated from the brain of a diseased horse in 1938 and has since caused periodic outbreaks across Central and South America. The VEEV species is classified into antigenic varieties such as IAB, IC, ID, and IE, all of which cause human disease that is indistinguishable between the antigenic varieties. Based on their epidemiological features and pathogenicity for equids, VEEV strains are categorized into two main ecological classes: epizootic/epidemic and enzootic/endemic. The epizootic strains, principally subtypes IAB and IC, have historically spread from South America as far north as Mexico and the southern United States, while enzootic strains, generally subtypes ID and IE, are typically confined to Central America and Mexico [3,4]. However, the geographic range of the ID subtype extends to South America, with notable outbreaks documented in Peru in 2006 and more recently between 2020 and 2021 [5].

In its ecological cycle, enzootic VEEV strains circulate continuously in forest and swamp habitats in a stable enzootic transmission cycle among small mammalian reservoir hosts (generally rodents) and mosquitoes of the genus *Culex*, specifically those belonging to the subgenus *Melanoconion* (denoted as *Culex* (*Melanoconion*) spp.), which serve as the primary enzootic vectors [6]. The emergence of epizootic strains from enzootic progenitors is primarily driven by critical adaptive mutations within the E2 envelope glycoprotein. These structural changes, often involving increased positive surface charge (e.g., mutations at E2 position 213), enhance the virus’s ability to infect epizootic mosquito vectors (such as *Aedes* and *Psorophora* spp.) and amplify efficiently in equine hosts. Consequently, when the virus evolves through these E2-specific mechanisms, its transmission dynamics change fundamentally. These epizootic strains are efficiently amplified in an equids–mosquito cycle, producing high titer viremia in horses that is sufficient for infection of susceptible mosquitoes. Subsequently, the virus spills over to infect humans through the bite of mammalophilic mosquitoes, including those in the *Aedes* and *Psorophora* genera, which can lead to extensive epidemics during epizootic amplification [7].

Human VEEV cases are characterized by a short incubation period of 1–5 days, followed by the onset of an acute febrile illness. Prominent clinical features include abrupt fever, rigors, severe cephalalgia, pain behind the eyes (retro-orbital pain), photophobia, and muscle pain, often accompanied by nausea and emesis. Mechanistically, this phase is driven by robust viral replication in peripheral lymphoid tissues, which triggers a massive systemic release of pro-inflammatory cytokines and chemokines (including IL-6, TNF-α, and IFN-γ) [8]. While this ‘cytokine storm’ is a hallmark of the host antiviral response, its dysregulation significantly contributes to acute morbidity. Although this acute phase is often self-limiting, the infection progresses to severe neurological disease in approximately 4% to 14% of cases. Notably, recent studies in non-human primate models indicate that even after physiological recovery from the acute phase, viral RNA and inflammation can persist in the CNS. While the overall case-fatality rate in humans is generally low (<1%), it can be significantly higher (up to 35%) in children with neurological symptoms. Survivors are frequently left with permanent neurologic sequelae, ranging from emotional instability and behavioral changes to severe outcomes such as seizures, motor impairment, and psychomotor retardation. In equids, the disease is far more lethal, with case fatality rates for epizootic strains ranging from 20% to 80% [9].

**Figure 1:**
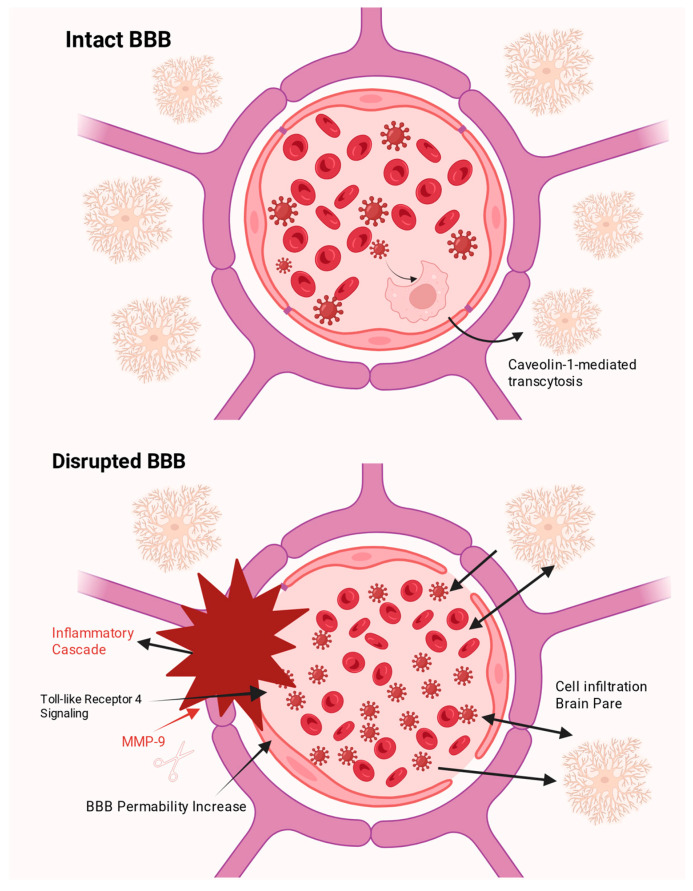
Hematogenous Route and Blood–Brain Barrier Disruption in VEEV Infection.

Following infection, VEEV invades the central nervous system (CNS) via two main pathways depending on the route of exposure. In cases of aerosol exposure or experimental intranasal inoculation, the virus utilizes the olfactory neuroinvasion pathway [10], spreading from olfactory sensory neurons to the olfactory bulb and subsequently to the cortex. Conversely, following a mosquito bite (the natural transmission cycle), the virus primarily enters via the hematogenous route. In this pathway, the virus circulates in the blood as free particles and crosses the intact blood–brain barrier (BBB) via Caveolin-1 (Cav-1)-mediated transcytosis across brain endothelial cells [11]. Significant increases in BBB permeability (or disruption) represent a secondary event, predominantly driven by an inflammatory cascade triggered by viral replication [12]. VEEV infection is known to activate Toll-like Receptor 4 (TLR4) signaling (and/or the TLR4 pathway), leading to the upregulation of inflammatory mediators such as matrix metalloproteinase-9 (MMP-9), which subsequently degrades tight junction proteins and compromises BBB integrity [13]. Furthermore, the peak of BBB permeability often coincides with the extravasation and infiltration of inflammatory monocytes, including CX3CR1^+^ and CCR2^+^ monocytes, into the brain parenchyma [14,15] (Figure 1).

This systematic review was conducted to synthesize current knowledge on VEEV structural biology, vaccine platforms, and therapeutic antibodies. To ensure rigor, we searched PubMed, Web of Science, and Google Scholar using keywords such as ‘VEEV structure’, ‘vaccines’, and ‘neutralizing antibodies’ for papers published through early 2025. We specifically included peer-reviewed studies on VEEV pathogenesis and clinical countermeasures while excluding studies with low thematic relevance.

## 2. Structural Characteristics

The biogenesis of VEEV structural components is a spatially coordinated process governed by the translational processing of the 26S subgenomic RNA. This mRNA is translated into a soluble polyprotein precursor, NH2-C-pE2(E3-E2)-6K-E1-COOH, which is co-translationally processed. The cycle initiates in the cytoplasm, where the Capsid protein (C) possesses serine protease activity and executes cis-autoproteolysis to release itself from the nascent polypeptide chain. The newly liberated Capsid rapidly recruits viral genomic RNA to self-assemble into the nucleocapsid core. Meanwhile, the remaining signal sequence at the N-terminus of E3 directs the translocation of the downstream envelope glycoprotein precursor (pE2-6K-E1) into the endoplasmic reticulum lumen.

Within the ER, host signalase performs trans-cleavages to separate pE2, 6K, and E1. Crucially, pE2 and E1 rapidly heterodimerize; in this complex, the E3 moiety functions as an intramolecular chaperone, stabilizing the heterodimer and masking the E1 fusion loop to prevent premature fusion during transport through the acidic compartments of the secretory pathway. Upon reaching the trans-Golgi network, the pE2 precursor undergoes a critical maturation step: it is cleaved by a host Furin-like protease into the mature E3 and E2 proteins. This cleavage destabilizes the E1–E2 interaction just enough to prime it for future fusion events. The final assembly is driven by a precise structural interaction at the plasma membrane: the cytoplasmic tail of E2 specifically inserts into a conserved hydrophobic pocket on the surface of the underlying nucleocapsid. This lock-and-key interaction provides the free energy required for the membrane to curve around the core, driving the budding of the infectious virion [16,17].

Given the central focus of this review on neutralizing antibodies, which primarily target these surface glycoproteins, the subsequent sections will detail the structural and functional characteristics of E1, E2, and Capsid, emphasizing their roles as targets for therapeutic intervention.

### 2.1. The E1 Glycoprotein

**Figure 2:**
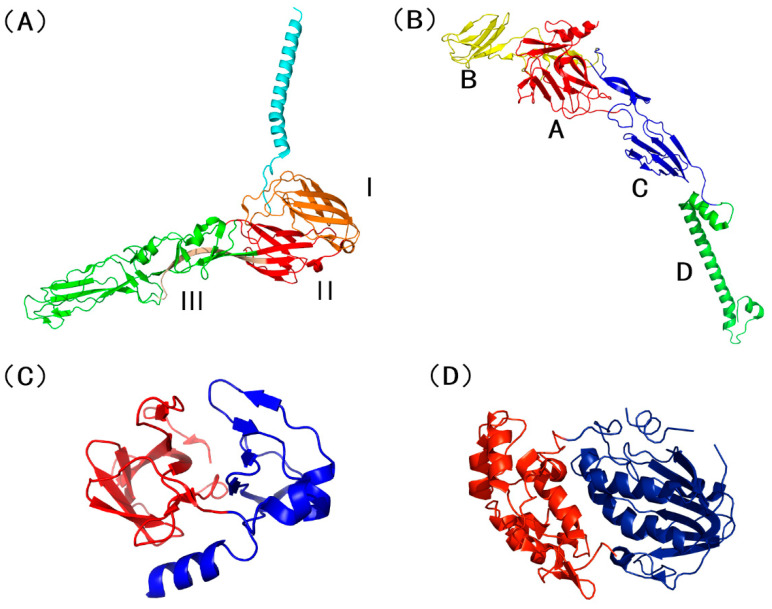
Schematic diagrams of VEEV key protein structures. (**A**) The E1 protein, showing domain I (orange), domain II (red), and domain III (green). (**B**) The E2 protein, showing domain A (red), domain B (yellow), domain C (blue), and domain D (green). (**C**) The capsid protein, showing the N-terminal region in red and the C-terminal domain in blue. (**D**) he nsP2 protein, showing the N-terminal domain in red and the C-terminal domain in blue.

The E1 glycoprotein, a class II fusion protein, is pivotal for alphavirus-mediated entry into the host cell, and its mature form possesses an approximate molecular weight of 48 kDa. The E1 ectodomain is an elongated molecule comprising three principal globular domains (DI, DII, and DIII), with its core structure being nearly entirely composed of β-sheet secondary structures. The central Domain I (DI) is interrupted by two extended insertions, which collectively constitute Domain II (DII). The core of DII is a short, six-stranded antiparallel β-sheet, from which the hydrophobic fusion loop is attached at the tip via the c-d and i-j loops. Domain III (DIII) exhibits an immunoglobulin-like fold and is connected to DI via a linker region [18] (Figure 2A).

During the virus assembly stage, proper folding and maturation of the E1 protein must occur within the endoplasmic reticulum (ER). Studies confirm that this process strictly depends on host chaperone systems, as the E1 protein has a direct physical interaction with host protein disulfide isomerase (PDI) family members, specifically PDIA6 (Protein Disulfide Isomerase A6). Mechanistically, the correct folding and functional integrity of E1 relies on the oxidoreductase activity of the PDI family to catalyze the formation of critical internal disulfide bonds. When PDI function is pharmacologically inhibited, E1 disulfide bond formation is blocked, leading to misfolding. Although virus particles are still released, these particles have lost infectivity, resulting in a large production of non-infectious viral particles. This demonstrated dependence on the host PDI system positions of E1 as an attractive, broad-spectrum antiviral drug target [19].

The mature E1 and E2 glycoproteins are tightly associated on the viral surface, forming a heterodimer. E1 is held in a metastable, prefusion conformation while E2 functions to mask the E1 fusion loop. Once the virus enters the host cell via endocytosis, the low pH (ranging from 5.5 to 6.0) of the endosomal compartment serves as the core signal to trigger E1 function. The acidic environment induces the dissociation of the E1–E2 heterodimers, which exposes the fusion loop of E1, allowing it to insert into the host endosomal membrane—a process that is highly dependent on cholesterol. Subsequently, three E1 monomers rapidly rearrange to form a stable homotrimer. Finally, E1 executes a hairpin-like conformational change, in which the distal DIII and stem region fold back against the trimer core, providing the mechanical force to drive the fusion of the viral membrane with the host membrane and release the viral genome [20].

Furthermore, the regulatory role of the *E1* gene extends to the level of RNA secondary structure, enabling a cell-type-specific replication control function independent of the protein coding sequence. It has been found that altering synonymous mutation sites within the *E1* gene, while keeping the E1 protein amino acid sequence identical, significantly enhances the virus’s replication fitness in macrophages. This effect is independent of the Type I interferon pathway. The underlying mechanism is that the altered RNA structure is able to specifically recruit host RNA-binding proteins (RBPs), which in turn upregulates the translational efficiency of the viral RNA. This discovery suggests that the RNA structure within the *E1* gene is a crucial non-coding element influencing viral replication and pathogenesis in macrophages [21].

### 2.2. The E2 Glycoprotein

The E2 glycoprotein (approximately 47 kDa) is a crucial component of the VEEV spike complex, where it is positioned atop the E1 protein and forms the distal tip of the spike. Traditionally viewed as the primary protein responsible for mediating receptor binding and eliciting the host neutralizing antibody response, E2 is considered the “first door” of virus–host interaction. Its structure encompasses three main globular domains (A, B, and C), with Domain B located farthest from the viral membrane. Additionally, VEEV E2 features a newly identified D-subdomain in its structure [22] (Figure 2B).

In the mechanism of viral entry, the function of E2 is more intricate than simple receptor binding, demanding precise synergy with the E1 protein. High-resolution structural studies have confirmed that the E2 glycoprotein functions cooperatively with E1 during viral entry, orchestrating the initial attachment and subsequent fusion events. Furthermore, the E2 D-subdomain acts as a critical “sensor” during endosomal acidification: this region, which is rich in conserved histidine residues, becomes protonated in the low pH environment, causing E2 to anchor to the endosomal membrane. This step is considered necessary to facilitate the dissociation of the E1–E2 dimer and expose the E1 fusion function [23].

The highly exposed nature of the E2 protein makes it the main target of the host immune system, particularly neutralizing antibodies (NAbs), and its sequence variation directly shapes viral pathogenicity and host range. Research profiling the human antibody response against VEEV has revealed that highly potent human NAbs predominantly recognize a novel functional neutralization epitope located in the E2 amino acid (aa) 115–119 region. This discovery holds significant clinical value: first, this “human epitope” is distinct from the previously identified major neutralization domain at E2 aa 182–207 found in murine models, offering new target combinations for developing “cocktail” antibody therapies that can evade viral immune escape. Second, the primary structural determinant of attenuation in the VEEV live-attenuated vaccine strain TC-83 is a single T120R mutation in the E2 protein, a site located proximal to this human neutralization epitope [24].

The recent identification of Low-Density Lipoprotein Receptor Class A Domain-Containing 3 (LDLRAD3) as the critical entry receptor for VEEV has provided profound insights into viral tropism and pathogenesis. High-resolution cryo-EM structures and mutagenesis studies reveal that the host LDLRAD3 engages the virus through its D1 domain—a cork-shaped structure containing a conserved calcium-binding cage—which binds cooperatively to the cleft formed between adjacent VEEV E2 and E1 glycoproteins within the trimeric spike. This high-affinity interaction involves key contacts between LDLRAD3-D1 residues (e.g., Trp47, Asp50, Asp54) and the E2 domain B, as well as the fusion loop of E1, effectively locking the fusion peptide to prevent premature membrane fusion. Receptor specificity is a critical determinant of host range and pathogenesis in encephalitic alphaviruses. Distinct differences exist in their entry mechanisms: whereas VEEV (encompassing subtypes IAB, IC, and ID) relies exclusively on LDLRAD3, recent genome-wide CRISPR screenings have revealed that both Western Equine Encephalitis Virus (WEEV) and Eastern Equine Encephalitis Virus (EEEV) predominantly utilize the Very-Low-Density Lipoprotein Receptor (VLDLR). Notably, WEEV is further distinguished by its engagement of Protocadherin-10 (PCDH10). Unlike LDLRAD3, VLDLR promotes infection of multiple members of the WEE complex but does not support VEEV entry, highlighting a strict receptor specificity. These structural and functional distinctions are paramount for therapeutic development; e.g., potent neutralizing antibodies could mimic the receptor interactions or by sterically occluding the receptor binding footprints on the E2 glycoprotein, thereby blocking viral attachment and entry [25,26].

Finally, E2 also plays an indispensable role in the later stages of the viral life cycle, specifically virus assembly and budding. The cytoplasmic tail of E2 passes through the viral envelope to interact directly with a hydrophobic pocket on the surface of the nucleocapsid core protein (CP). This E2-CP interaction across the membrane provides the key mechanical force that drives the correct assembly of nascent virions, allowing them to acquire T = 4 icosahedral symmetry and bud successfully from the host cell membrane. The stability of this connection is reinforced by conserved, potentially palmitoylated cysteine residues in the E2 tail anchored to the inner leaflet of the plasma membrane [18].

### 2.3. The Capsid Protein

The Venezuelan equine encephalitis virus Capsid Protein (CP) is a fundamental structural unit that forms the virus core. The capsid protein is primarily responsible for packaging the viral 11.5 kb RNA genome. It subsequently self-assembles to form the nucleocapsid (NC) core with T = 4 icosahedral symmetry, thereby encasing the viral genome and protecting it from degradation. VEEV CP is composed of 275 amino acids and can be functionally divided into the N-terminal domain (approximately aa 1 to 126) and the C-terminal domain (approximately aa 127–275). The N-terminal domain is notably rich in positively charged amino acids and is highly basic, showing an unstructured or disordered state, which is believed to extend into the nucleocapsid interior to interact with genomic RNA via electrostatic interactions. This N-terminal region can be further divided into four structural subdomains (SDs): SD1 (aa 1 to 37) was found to be dispensable for particle formation but critically important for VEEV capsid function12. SD2 (aa 38 to 51) contains a small alpha helix (Helix I) 14, and in VEEV it functions as a nuclear export signal (NES). SD3 (aa 52 to 110) is characterized as a highly positively charged subdomain and the most positively charged capsid-specific subdomain, primarily responsible for RNA-capsid protein binding, while also functioning as a negative regulator of NC and particle assembly, being only effectively assembled until bound to viral genomic RNA. SD4 (aa 111 to 126) contains a highly conserved sequence and is suggested to determine the specificity of viral genome packaging. Connecting the N-terminal and C-terminal domains is a linker region (aa 109–125 for VEEV), which includes a stretch of highly conserved residues that binds to cellular 60S ribosomal subunits, mediating the disassembly of nucleocapsid cores after they are released into the cytosol. The CP C-terminal domain, in contrast, folds into a chymotrypsin-like fold and harbors a serine proteinase active site that facilitates the autoproteolytic processing (self-cleavage) of CP during structural polyprotein synthesis. Furthermore, the C-terminal domain contains a hydrophobic pocket that serves as the crucial binding site for the cytoplasmic domain of the E2 envelope glycoprotein. Structural studies on the closely related prototype alphavirus, Sindbis virus (SINV), have revealed that specific hydrophobic residues (E2-400 to E2-402) insert into this pocket. Due to the high structural conservation among alphaviruses, this ‘lock-and-key’ interaction is considered the universal mechanism driving nucleocapsid-glycoprotein envelopment in VEEV and other faminly members [27] (Figure 2C).

Beyond its foundational role as a structural element, VEEV CP also exhibits critical nonstructural functions in the host–virus conflict and is considered one of the major determinants of virulence. Research has demonstrated that VEEV capsid protein contains both a nuclear localization signal (NLS) (located within SD3) and a nuclear export sequence (NES) (located within SD2), allowing it to shuttle between the cytoplasmic and nuclear compartments of infected cells. Research has established that the Very-Low-Density Lipoprotein Receptor (VLDLR) serves as a primary entry mediator for both Western Equine Encephalitis Virus (WEEV) and Eastern Equine Encephalitis Virus (EEEV). Additionally, WEEV has been shown to specifically exploit Protocadherin-10 (PCDH10) as a distinct receptor. This capsid-induced block in nucleocytoplasmic trafficking results in transcriptional suppression and an overall shutdown of host transcription. Further studies indicate that this transcriptional downregulation directly impacts cell cycle regulation, causing infected cells to stall or halt the cell cycle in G0/G1 phase. Mechanistically, the capsid’s nuclear localization is necessary, leading to the downregulation of key cell cycle regulators (including Cyclin D1, Cyclin A2, and Cyclin E2) and suppression of Rb phosphorylation. Infection using an NLS mutant VEEV was partially rescued from this cell cycle dysregulation and showed a less pronounced decrease in host transcriptional expression, highlighting the importance of capsid nuclear localization and/or importin α binding for inducing cell cycle arrest and solidifying the central role of CP’s nuclear functions in VEEV pathogenesis. Thus, the VEEV CP is not merely a structural foundation but a “clever” multifunctional protein that enhances viral replication and spread by hijacking the host’s nuclear transport system [28].

### 2.4. The 6K Protein

The structural polyprotein ORF of the alphavirus genome contains a coding sequence for the small hydrophobic protein 6K, situated between the *E2* and *E1* genes. This protein is named for its approximate 6 kDa molecular weight and is classified as a member of the viroporin family. Traditionally, the 6K protein has been considered a component involved in membrane permeabilization. Structurally, 6K is a transmembrane protein rich in cysteine residues. Models regarding its membrane topology vary: earlier hypotheses suggested it possessed two transmembrane domains (TMD), while newer data posits it may contain only a single TMD [29].

The long-observed phenomenon of the 6K protein appearing as double bands on gels has been resolved by recent investigations: a highly conserved “slippery” sequence, UUUUUUA, exists within the 6K coding sequence, which induces programmed -1 ribosomal frameshifting at an efficiency of 10% to 18%. This translational event yields two distinct proteins: the canonical 6K protein, translated in the standard reading frame, and the TransFrame protein (TF), an ~8 kDa transmembrane protein produced via frameshifting with a unique C-terminal sequence [30].

Further research demonstrates that both 6K and TF proteins play interconnected yet specialized roles in the viral life cycle. The canonical 6K protein’s primary function centers on polyprotein processing, with its C-terminal domain serving as a signal peptide essential for the downstream E1 glycoprotein to enter the endoplasmic reticulum (ER). Furthermore, 6K functions as a viroporin in internal membrane systems, potentially aiding glycoprotein transport or facilitating the appropriate ionic environment for budding by forming an ion channel. Although deletion of 6K (and TF) in various alphaviruses (like SFV and SINV) substantially reduces virus titer, its absence in some strains, such as Salmonid Alphavirus (SAV3), renders the resulting virus non-viable [31].

In contrast, the TF protein assumes a more crucial and direct role in the late stage of the viral life cycle: assembly and budding. The TF protein’s unique C-terminal sequence is extensively modified by cellular enzymes through palmitoylation, an event hypothesized to be the key signal that targets the protein specifically to the plasma membrane. Mass spectrometry and immunological assays confirm that TF, and not the 6K protein, is predominantly incorporated into mature virions. TF facilitates the efficient assembly and detachment of progeny particles by directly regulating membrane curvature, interacting with envelope proteins like E2, and possibly recruiting specific lipids. Deletion or mutation of TF palmitoylation sites frequently leads to reduced viral budding efficiency and the potential formation of aberrant multinucleocapsid particles. Thus, the TF protein produced via programmed frameshifting is an important regulatory factor for efficient viral replication and mature virion formation [32].

## 3. Vaccine

Vaccination remains the paramount prophylactic strategy against VEEV infection. Leveraging the structural insights of the E1 and E2 glycoproteins previously discussed, the primary goal of vaccine development is the safe and efficacious induction of high-titer neutralizing antibodies (NAbs), which are crucial for long-term protection. While NAbs are the principal immunological correlate of efficacy, optimizing comprehensive immune responses, including cellular immunity, is also a focus. However, the field is challenged by the lack of a formally licensed vaccine for human use. The historical live-attenuated TC-83 strain, used only under Investigational New Drug (IND) status, exhibits high reactogenicity and suboptimal seroconversion rates. Consequently, next-generation vaccine efforts are focused on diverse platforms—such as live-attenuated, VLP, recombinant subunit, and nucleic acid vaccines—to engineer formulations capable of eliciting broader and more durable protective immunity [33].

### 3.1. Inactivated Vaccines

Inactivated vaccines aim to completely eliminate viral infectivity through physical or chemical means while retaining immunogenic structures, thereby offering a superior safety profile compared to live-attenuated vaccines. The historical standard, C-84 (formalin-inactivated TC-83), was primarily used to boost non-responders to TC-83; however, it is limited by short-lived immunity, the requirement for multiple doses, and an inability to induce effective mucosal immunity against aerosol challenge [34]. To overcome these deficiencies, contemporary research has shifted toward utilizing genetically engineered attenuated strains, such as V3526, to ensure safety even in the event of incomplete inactivation. Comparative studies by Fine et al. demonstrated that while heat inactivation effectively eliminates infectivity, it denatures critical neutralizing epitopes on E2/E3 glycoproteins, resulting in a loss of antibody binding capacity; similarly, high-dose gamma irradiation, though primarily targeting nucleic acids, also compromises antigen binding. In contrast, optimized formalin inactivation (fV3526) superiorly preserves neutralizing epitopes and confers complete protection against lethal subcutaneous and aerosol challenges in mice [35]. Furthermore, Martin et al. confirmed that fV3526, when formulated with novel adjuvants, elicits potent protective immunity at lower antigen doses than C-84. Additionally, Sharma et al. proposed a novel inactivation strategy using the hydrophobic photoactive compound 1,5-iodonaphthyl azide (INA), which selectively binds to the hydrophobic domains of transmembrane proteins; this approach effectively inactivates the virus while preserving the structural integrity of the E1 and E2 glycoprotein ectodomains, thus maximally maintaining native conformation and immunogenicity [36].

### 3.2. Live-Attenuated Vaccines

Live-attenuated vaccines are designed to elicit comprehensive and durable immunity by attenuating viral pathogenicity while preserving replicative competence. Given the established limitations of the traditional TC-83 vaccine, such as reactogenicity and partial non-responsiveness, Pratt et al. engineered the next-generation candidate V3526 via site-directed mutagenesis by deleting the PE2 furin cleavage signal and incorporating a suppressor mutation at E1-253; this construct demonstrated avirulence in animal models and robust protection against aerosol challenge. Expanding on this efficacy profile, Reed et al. substantiated that V3526 confers solid cross-protection in non-human primates not only against homologous IA/B strains but also against aerosol exposure to the genetically divergent virulent subtype IE strain (68U201), outperforming the subtype-specific candidate IE1150K [37,38].

Regarding attenuation mechanisms and safety enhancement, Garmashova et al. identified the N-terminal domain of the viral capsid protein as the determinant for transcriptional shutoff; modifying this region via substitution or frameshift mutations successfully abrogated cytopathogenicity and reduced in vivo virulence while maintaining viral replication [39]. Building upon the V3526 platform, Haines et al. recently introduced high-fidelity mutations (3X and 4X) into the RNA-dependent RNA polymerase (RdRp), which restricted tissue tropism—notably in the spleen and kidneys—thereby enhancing the safety profile without compromising immunogenicity [40]. Furthermore, addressing manufacturing and biosafety challenges, Samsa et al. developed the LAV-CNE vaccine utilizing a cationic nanoemulsion to deliver the synthetic RNA genome of TC-83; this self-amplifying RNA (SAM) approach initiated a viral replication cycle in vivo, eliciting protective immunity equivalent to live virus vaccination while circumventing the risks associated with traditional live virus production [41].

### 3.3. Recombinant Vaccines

Given the safety concerns associated with live virus vaccines and the immunogenicity limitations of traditional inactivated vaccines, researchers have turned to recombinant viral vector technology to develop safer and more effective VEEV vaccines. Paessler et al. employed a chimeric virus strategy, replacing the structural protein genes of the non-pathogenic Sindbis virus (SINV) with those of VEEV TC-83 (C-E3-E2-6K-E1) to construct the SIN-83 chimeric vaccine. In mouse models, this vaccine not only demonstrated superior safety compared to TC-83 (absence of neurotoxicity) but also induced potent neutralizing antibodies and conferred complete protection against lethal virulent VEEV challenge following a single immunization [42]. Guerbois et al. further explored regulating viral protein expression using Internal Ribosome Entry Sites (IRES), constructing the VEEV/IRES/C vaccine candidate. By placing the *capsid* gene under the control of the encephalomyocarditis virus (EMCV) IRES, this strategy maintained efficient replication in vertebrate cells while abrogating mosquito transmission due to the IRES’s inactivity in insect cells, thereby eliminating the ecological risk of natural spread while preserving immunogenicity and protective efficacy [43].

Furthermore, addressing the suboptimal immunogenicity of adenovirus-vectored vaccines, Williams et al. utilized codon optimization technology to significantly enhance the expression of VEEV E3-E2-6K structural proteins in mammalian cells. The optimized adenovirus vaccine elicited approximately 10-fold higher VEEV-specific antibody titers in mice compared to the non-optimized control and provided significantly enhanced protection against lethal aerosol challenge [44]. Tretyakova et al. developed a DNA-launched live-attenuated vaccine platform, constructing the genetically rearranged V4020 vaccine strain. V4020 incorporates TC-83 attenuation mutations and prevents reversion to virulence through structural gene rearrangement [45]. Delivered as a DNA plasmid in non-human primates, this vaccine initiated a limited round of viral replication in vivo, inducing robust neutralizing antibodies and completely blocking viremia following aerosol challenge, with high genetic stability confirmed by deep sequencing. These recombinant vaccine strategies demonstrate the immense potential of genetic engineering to precisely modulate viral replication, expression, and transmission, achieving an optimal balance between safety and efficacy.

### 3.4. Nucleic Acid Vaccines

Nucleic acid vaccines, particularly DNA vaccines, represent a revolutionary strategy in vaccinology. The fundamental principle involves delivering plasmid DNA encoding pathogen antigens directly into host cells, utilizing the host’s transcriptional and translational machinery to synthesize antigens in situ. This process enables antigen presentation via both MHC class I and II pathways, simultaneously eliciting humoral and cellular immunity. Compared to traditional vaccines, DNA vaccines offer significant advantages, including low production costs, high thermal stability, lack of infectious risk, and ease of multivalent construction; however, the immunogenicity of naked DNA in large animals and humans is often suboptimal, necessitating physical delivery methods, such as electroporation, to enhance transfection efficiency [46].

Regarding VEEV, Riemenschneider et al. demonstrated the compatibility of DNA vaccines in multivalent formulations, finding that a combined DNA vaccine containing antigens for VEEV, *Bacillus anthracis*, Ebola, and Marburg viruses showed no immune interference [47]. Dupuy et al. utilized intramuscular electroporation (IM-EP) to deliver a trivalent DNA vaccine encoding the E2-E1 glycoproteins of VEEV, EEEV, and WEEV; this approach induced robust and durable neutralizing antibodies in non-human primates and conferred complete protection against aerosol challenge, validating the efficacy of electroporation [48]. Addressing the logistical limitations of electroporation devices, Suschak et al. evaluated needle-free jet injection (NFJI) and found that the pWRG/VEE vaccine delivered via the PharmaJet Stratis device elicited protective immune responses in non-human primates comparable to electroporation, offering a more practical alternative for mass deployment [49]. In terms of vector optimization, Suschak et al. also enhanced the immunogenicity of VEEV DNA vaccines without increasing the dose by using Nanoplasmid vectors co-expressing innate immune agonists. Furthermore, Tretyakova et al. developed an innovative “iDNA” vaccine strategy, where plasmid DNA transcribes infectious, attenuated viral RNA (V4020) in vivo. This “DNA-launched live-attenuated vaccine” combines the genetic stability of DNA vaccines with the high potency of live vaccines, conferring complete protection in mice after a single dose [50].

### 3.5. Virus-like Particle Vaccines

Virus-like particle (VLP) vaccines mimic the morphological conformation and antigen presentation of native viruses through the self-assembly of viral structural proteins but are entirely non-infectious due to the absence of the viral genome; this unique structural characteristic confers both a high safety profile and potent immunogenicity, particularly as the repetitive arrangement of conformational epitopes on the particle surface can effectively cross-link B cell receptors to induce high-titer neutralizing antibodies and cellular immunity [51]. Addressing VEEV protection, Ko et al. engineered a trivalent VLP vaccine (WEVEE) incorporating the structural proteins (C-E3-E2-6K-E1) of VEEV, EEEV, and WEEV; this candidate induced balanced and robust neutralizing antibody responses against all three viruses in non-human primates and conferred complete protection against lethal aerosol challenge, with no evidence of immune interference among the antigenic components [52]. Building upon these preclinical data, Ee et al. conducted a first-in-human Phase 1 clinical trial of this trivalent VLP vaccine, confirming its favorable safety and tolerability profile in healthy adults while inducing durable specific neutralizing antibody responses, thereby validating the clinical translational potential of the VLP platform for equine encephalitis virus vaccines [53].

## 4. Functional Antibodies

Among the VEEV structural proteins, the surface-exposed glycoproteins E1 and E2, which are essential for mediating viral entry into host cells, represent the principal targets for the host B cell response and neutralizing antibodies (NAbs). The development of antibodies capable of blocking the function of these proteins has become a central strategy in VEEV-related prophylaxis and therapy. This section will review the research progress of representative anti-VEEV antibodies, tracing their development from the humanization of early murine antibodies to the application of novel platforms and computational design for next-generation biologics.

### 4.1. Humanization and Functional Validation of Murine Antibodies

Early investigations into VEEV antibodies centered on murine monoclonal antibodies (mMAbs), which validated the potential for antibody-mediated protection in animal models. However, the inherent immunogenicity of these xenogeneic proteins in humans, which can elicit a human anti-mouse antibody response, necessitated their humanization for clinical consideration.

#### 4.1.1. Humanized 3B4C-4 Antibody (Hy4 IgG)

The murine mAb 3B4C-4, a potent neutralizing antibody targeting the VEEV E2 glycoprotein E2c epitope, was successfully humanized by Hunt et al. [54] using combinatorial antibody libraries and phage-display technology, yielding Hy4 IgG. In vitro characterization, including Plaque Reduction Neutralization Tests (PRNT), confirmed that Hy4 IgG retained a virus-neutralizing capacity (70% PRNT endpoint of 39 ng/mL) comparable to, or even exceeding, its murine parent 3B4C-4 (100 ng/mL). The antibody demonstrated substantial in vivo protective efficacy in Swiss Webster mice. Prophylactically, a dose as low as 100 ng provided 90% protection against an intraperitoneal challenge administered 24 h later, while a 500 µg dose protected 80% of mice from an intranasal challenge. Furthermore, its therapeutic potential was established, as administration of Hy4 IgG cured 90% of mice when given within 1 h of i.p. infection and 75% of mice when treatment was delayed to 24 h post-infection.

#### 4.1.2. Humanized 1A4A1 Antibody (hu1A4A1IgG1-2A)

The 1A4A1 murine antibody, also targeting the E2c epitope, was humanized by Hu et al. using Complementarity-Determining Region (CDR) grafting. An initial construct utilizing a furin-cleavable linker failed to process correctly in mammalian cells. A subsequent, optimized construct, hu1A4A1IgG1-2A, successfully employed a foot-and-mouth-disease virus (FMDV)-derived 2A self-cleavage oligopeptide, which resulted in complete and proper cleavage of the heavy and light chains. This engineered antibody retained in vitro characteristics comparable to its murine parent, including a similar antigen-binding affinity and an identical virus-neutralizing activity. In a Balb/c mouse model, hu1A4A1IgG1-2A demonstrated potent prophylactic and therapeutic efficacy, with a single 50 µg dose providing 100% protection against lethal subcutaneous challenge when administered either 24 h before or 24 h after virus inoculation. However, this therapeutic window was narrow, as protection was lost when treatment was delayed to 72 h post-infection [55,56].

#### 4.1.3. Humanized and Chimeric 1A3B-7 Antibodies (Hu1A3B-7/c1A3B-7)

The murine antibody 1A3B-7 has garnered significant interest due to its broad specificity, capable of recognizing multiple VEEV serogroups. Goodchild et al. [57] employed a CDR grafting strategy to develop a humanized version, Hu1A3B-7. In vitro characterization, including ELISA and plaque assays, confirmed that Hu1A3B-7 retained the broad specificity and neutralizing activity of its murine parent. This construct successfully protected mice against lethal subcutaneous and aerosol challenges. Furthermore, Burke et al. evaluated a murine-human chimeric version, c1A3B-7, in a nonhuman primate model. While c1A3B-7 demonstrated superior antigen binding in ELISA and Bio-Layer Interferometry assays compared to hu1A4A1-YTE, it showed substantially weaker in vitro neutralization in PRNT assays (~20% neutralization at 6 ng/mL vs. >90% for hu1A4A1-YTE). Despite this, c1A3B-7 provided superior therapeutic efficacy in NHPs following aerosol exposure, significantly reducing viremia and lymphopenia when administered 24 h post-exposure. Protection was even observed when treatment was delayed to 48 h post-exposure.

### 4.2. Exploration of Novel Epitopes and Antibody Functions

Research has expanded beyond conventional E2 targets to identify novel protective epitopes and to understand the role of non-neutralizing antibodies, revealing a more complex landscape of immune protection. To provide a structural framework for the diverse antibody candidates discussed throughout this review, Table 1 summarizes their specific targets and epitopes, with a particular focus on their spatial relationship to the critical LDLRAD3 receptor-binding site and the E1 fusion loop.

#### 4.2.1. Protective Antibodies Targeting the E3 Glycoprotein (13D4)

Parker et al. [58] reported a significant finding by isolating a group of murine mAbs, represented by 13D4, that specifically target the E3 glycoprotein. These antibodies were generated using a V3526 furin cleavage site deletion mutant. Functionally, 13D4 neutralized the V3526 mutant virus but did not neutralize the parental wild-type (WT) VEEV TrD in a standard PRNT. However, in virus yield assays, 13D4 dramatically inhibited the production of infectious virus from WT VEEV TrD-infected cells by up to 1000-fold. This in vitro inhibition of viral budding translated to in vivo efficacy, as passive immunization with 13D4 provided significant protection to mice against a lethal VEEV TrD challenge. This study was the first to identify a protective epitope on the E3 glycoprotein, opening a new avenue for therapeutic design.

#### 4.2.2. Broad-Spectrum, Non-Neutralizing Antibody (CUF37-2a)

To derive broadly reactive antibodies, O’Brien et al. [59] immunized mice with a mixture of VEEV strains (serotypes I-VI) and constructed an scFv phage display library. A highly cross-reactive scFv was identified and converted into a murine IgG2a, designated CUF37-2a. ELISA analysis confirmed its broad reactivity against VEEV subtypes I-V. Despite its ability to bind the E2 glycoprotein, CUF37-2a was found to be non-neutralizing in vitro when tested against multiple subtypes in a plaque assay. Nevertheless, prophylactic administration of CUF37-2a 24 h prior to challenge provided significant, dose-dependent protection in mice, with a 50% protective dose (PD50) estimated at 9.15 µg. This work provided clear evidence that in vivo protection is not exclusively dependent on in vitro neutralization, suggesting a critical role for Fc-mediated effector functions.

#### 4.2.3. Human Neutralizing Antibody F5

Hunt et al. [60] described F5 nIgG, a fully human monoclonal antibody isolated via phage display from a VEEV TC-83-vaccinated donor. F5 targets a novel, highly potent neutralization epitope within the E2 domain A (amino acids 115–119). It demonstrated strong in vitro neutralization, with a 70% plaque-reduction (PRNT70) endpoint of 12.5 ng/mL. In mouse models, F5 nIgG provided robust protection when administered prophylactically 24 h before either subcutaneous or aerosol challenge. Furthermore, it showed therapeutic efficacy; when administered 24 h after a lethal aerosol challenge, mice were protected from clinical disease and mortality. Although immunohistochemistry (IHC) and qRT-PCR confirmed high viral titers in the brains of these treated mice at early time points, the animals remained asymptomatic and successfully cleared the virus from the CNS by 14–28 days post-infection.

#### 4.2.4. Human Polyclonal Antibodies from Transchromosomic Bovines

Addressing the need for a scalable source of human antibodies, Gardner et al. [61] utilized a transchromosomic bovine platform. These animals are engineered to produce fully human polyclonal antibodies (PAbs). Hyperimmunization of Tc bovines with an AMT-inactivated wild-type TrD-derived virus (V3000 nt3A) generated high-titer, VEEV-specific human PAb preparations (TcPAbs). These TcPAbs were potently neutralizing in an Infectivity Reduction Neutralization Test (IRNT), with V3000 nt3A AMT-derived antisera reaching titers of ~1:14,000. In murine models, these TcPAbs provided robust protection against lethal subcutaneous and aerosol VEEV challenges in both prophylactic and therapeutic settings.

#### 4.2.5. Vaccine-Elicited Broadly Protective Antibody (SKT05)

Sutton et al. [62] investigated the antibody repertoire induced by a trivalent VLP vaccine (WEEV, EEEV, and VEEV) in macaques. This regimen elicited a diverse B cell response, including triple-specific antibodies. One such antibody, SKT05, demonstrated potent neutralization of all three EEV pseudoviruses in an Env-pseudotyped lentiviral reporter assay. Cryo-EM structural analysis revealed a unique mechanism for its breadth: SKT05 targets a conserved epitope on the E1 protein, proximal to the fusion peptide, and engages backbone atoms of sequence-diverse residues to accommodate variability. Notably, SKT05’s protective capacity extended in vivo to both encephalitic (VEEV) and arthritogenic (CHIKV and RRV) alphaviruses, demonstrating remarkable cross-clade protection.

#### 4.2.6. Panel of Neutralizing Murine and Human mAbs

Kafai et al. [63] characterized a new panel of murine and human neutralizing mAbs from subjects immunized with attenuated VEEV strains. Using ELISA-based competition binding assays, these mAbs were classified into three distinct antigenic groups (Groups I, II, and III), targeting epitopes in the A or B domains of the E2 glycoprotein. Detailed functional assays revealed that these antibodies inhibit multiple steps of the viral lifecycle. The human mAb hVEEV-63 (Group II) was found to be exceptionally potent, with an FRNT50 < 2 ng/mL against SINV-VEEV TrD. A 3.2 Å cryo-EM reconstruction confirmed its binding to the E2 domain B and suggested a neutralization mechanism involving the cross-linking of neighboring spikes. These mAbs provided robust prophylactic and therapeutic protection against lethal VEEV aerosol challenge in mice.

### 4.3. Antibody Engineering, Optimization, and Computational Design

Recent research has focused on enhancing antibody performance through bioengineering and applying computational methods to accelerate discovery and optimization.

#### 4.3.1. Bivalent Single-Domain Antibodies (sdAbs)

To address the size and stability limitations of conventional IgG, Liu et al. [64] developed single-domain antibodies from a phage display library derived from a llama immunized with a VEEV-containing equine vaccine. Monovalent sdAbs like V3A8 and V2B3 showed moderate neutralization in PRNT assays (PRNT50 of 0.16 µg/mL and 0.95 µg/mL, respectively). The key innovation was the creation of bivalent constructs; genetically linking two sdAbs improved neutralizing potency by up to two orders of magnitude. The hetero-bivalent construct V2B3-V3A8f was exceptionally potent, achieving a PRNT50 of 0.003 µg/mL against the virulent VEEV TrD strain, highlighting the therapeutic potential of engineered sdAb formats.

#### 4.3.2. Computational Affinity Maturation of F5 (SNL1-1)

Sumner et al. [65] combined computational modeling with experimental library screening to affinity-mature the F5 antibody. Despite the challenge of modeling F5’s long 20-amino-acid H3 loop, Rosetta and dTERMen software were used to predict affinity-enhancing mutations. A phage display library screening these predictions yielded an optimal variant, SNL1-1. BLI analysis confirmed a 63-fold improvement in binding affinity (KD reduced from 0.63 nM to 0.01 nM), primarily driven by a 60.3-fold slower dissociation rate (kd). While this did not enhance the PRNT neutralization potency against VEEV-TC83, Gyrolabs immunoassays showed it significantly increased cross-reactive binding to other VEEV subtypes, such as IC and IIIA.

#### 4.3.3. Interplay Between Fc Effector Functions and Binding Potency

To dissect the mechanisms of antibody protection in vivo, Schwedler et al. [66] engineered the F5 antibody to create an effector-null variant, hF5-LALA (L234A/L235A). A suite of in vitro assays confirmed that the LALA mutations, while preserving antigen binding and neutralization, significantly reduced binding to C1q and FcyRs. Functional assays, including RT-CDC and RT-ADCC (real-time cytotoxicity assays), showed delayed CDC kinetics and a complete abrogation of ADCC activity for hF5-LALA. When tested in vivo in a C3H/HeN mouse model, both hF5-WT and hF5-LALA provided high-level protection when administered early (at −24 h or +24 h post-infection). However, when treatment was delayed to +48 h post-infection, hF5-LALA’s efficacy significantly decreased (51.4% survival) compared to the hF5-WT (85.7% survival). This study provides definitive evidence that while neutralization is sufficient for early intervention, optimal therapeutic efficacy in an established VEEV infection is contingent upon intact Fc effector functions.

However, a recent study by Callahan et al. [67] adds a critical nuance to this paradigm by demonstrating that high binding potency can overcome the requirement for Fc effector functions. By comparing two broadly reactive monoclonal antibodies, SKT05 and SKT20, the authors found that while the lower-potency antibody (SKT20) strictly required Fc effector functions to prevent lethality, the high-potency antibody (SKT05) conferred survival independently of Fc functions. This Fc-independent protection by SKT05 was linked to its ability to robustly inhibit viral egress. Notably, the study established a dose-dependent relationship, showing that increasing the dosage of lower-potency antibodies could compensate for their lack of Fc effector functions. These findings suggest that while Fc-mediated clearance is critical for antibodies with moderate affinity, optimizing binding potency and egress inhibition represents an alternative strategy for developing next-generation therapeutics that are less reliant on host effector systems.

#### 4.3.4. Generative AI-Designed sdAbs (a18, a155)

In a novel application of artificial intelligence, Liu et al. [68] utilized a generative AI platform for the de novo design of anti-VEEV sdAbs. A ProtGPT2 language model was fine-tuned using a small dataset (n = 62) of known alphavirus-binding sdAbs. The AI-generated sequences were then screened in silico using AlphaFold2-multimer (for structure) and Prodigy (for affinity). Eight candidate sdAbs were selected for experimental validation. ELISA results confirmed that several candidates, notably a18 and a155, successfully bound to irradiated VEEV TC-83 particles. These AI-designed sdAbs also demonstrated modest, but measurable, virus-neutralizing activity in PRNT assays (PRNT50 values of ~39–44 µg/mL). This work provides a crucial proof-of-concept for using generative AI to accelerate the design of novel antibody therapeutics.

## 5. Conclusions and Outlook

Venezuelan equine encephalitis virus persists as a significant public health and biodefense concern. Our understanding has matured from static structural descriptions to a dynamic view of a multifunctional proteome. Key insights include the capsid’s non-structural role in host transcriptional suppression and the function of the frameshift-derived TF protein in viral budding. Concurrently, the identification of host factors like the LDLRAD3 entry receptor has clarified critical pathogenic mechanisms. In parallel, therapeutic strategies have evolved beyond the humanization of murine antibodies to the identification of novel protective epitopes on E1 and E3. A critical finding has emerged: optimal in vivo therapeutic efficacy, particularly post-exposure, is contingent not only on neutralization but also on intact Fc effector functions. The outlook is increasingly defined by innovative platforms, including potent engineered single-domain antibodies, scalable human polyclonal antibodies from transchromosomic bovines, and the pioneering use of generative AI for de novo antibody design. Future efforts must focus on optimizing these next-generation biologics, defining mechanisms of broad protection, and translating these advanced molecular insights into effective clinical countermeasures.

## Figures and Tables

**Table 1:** Summary of representative anti-VEEV antibodies and their binding characteristics relative to key structural motifs.

Antibody Name	Format/Type	Target Protein	Binding Domain	Structural Context
3B4C-4	Murine IgG	E2	Domain B (Tip)	Near LDLRAD3
1A4A-1	Murine IgG	E2	Domain B (E2c)	Near LDLRAD3
TRD-14	Murine IgG	E2	Domain B (Distal)	Neither
F5 (hF5)	Human IgG	E2	Domain A	Neither
SNL1-1	Human IgG	E2	Domain A	Neither
hVEEV-63	Human IgG	E2	Domain B	Near LDLRAD3
SKT05	Macaque IgG	E1	Fusion Loop	Near Fusion Loop
SKT20	Macaque IgG	E1	Fusion Loop	Near Fusion Loop
1A3B-7	Murine IgG	E2	E2	Unknown
CUF37-2a	Murine IgG	E2	E2	Neither
13D4	Murine IgG	E3	E3 Glycoprotein	Neither
V3A8	Llama sdAb	E2	E2	Unknown
a18	AI-Designed	E2	E2	Unknown
TcPAbs	Polyclonal	Various	Polyclonal	Mixed

## Data Availability

Not applicable.
